# The Promise of the Dual Prevention Pill: A Framework for Development and Introduction

**DOI:** 10.3389/frph.2021.682689

**Published:** 2021-06-23

**Authors:** Barbara A. Friedland, Sanyukta Mathur, Lisa B. Haddad

**Affiliations:** 1 Population Council, Center for Biomedical Research, New York, NY, United States,; 2 Population Council, HIV and AIDS Program, Washington, DC, United States

**Keywords:** HIV prevention, multipurpose prevention technologies, PrEP-FP integration, integrated healthcare, oral contraceptives, PrEP, dual prevention pill, informed choice

## Abstract

Women of reproductive age need multipurpose prevention technology (MPT) products to address two overlapping health risks: unintended pregnancy and HIV. Currently, condoms are the only available MPT, however male condoms are not within the control of a woman, and the use of female condoms has been limited by low acceptability and cost. Oral pre-exposure prophylaxis (PrEP) is highly effective for HIV prevention, yet uptake and adherence among women have been low to date. Women globally need more options for HIV and pregnancy prevention. Several MPTs for simultaneous HIV and pregnancy prevention are in various stages of development and clinical testing, although most are many years away from market launch. A dual prevention pill (DPP), a daily oral pill combining oral contraceptives and PrEP, both of which are licensed, approved products in many low- and middle-income countries (LMIC), is likely to be the fastest route to getting an MPT product into the hands of women. The DPP is one option that could enhance method choice, particularly for women who are already using oral contraceptives. By leveraging the oral contraceptive market and reaching women currently using condoms or with an unmet need for contraception, the DPP has the potential to increase the uptake of PrEP. The successful rollout of the DPP will require careful consideration of user-, provider-, and product-centered factors during product development and introduction. Early attention to these interrelated factors can help ensure that the DPP has the ideal characteristics for maximum product acceptability, that effective and quality services are designed and implemented, and that users can make informed choices, demand the product, and use it effectively. The proposed framework outlines key considerations for the effective development and introduction of the DPP, which could also facilitate integration models for future MPTs.

## INTRODUCTION

Women worldwide are confronted with two significant, overlapping health risks: unintended pregnancy and HIV/sexually transmitted infections (STIs). More than 218 million women in low- and middle-income countries (LMICs), including 26% of women in sub-Saharan Africa (SSA), have an unmet need for contraception (1). Although, significant advances have been made in HIV treatment and prevention over the last decade, HIV/AIDS continues to be a leading cause of death among women of reproductive age globally (2). Nearly 800,000 women aged 15 and above were newly infected with HIV in 2019 (2). In SSA, women and girls accounted for 59% of all new HIV infections, and adolescent girls and young women (AGYW) aged 15–24 years old were twice as likely to be living with HIV compared to their male counterparts (2). Despite substantial efforts by the global health community to integrate HIV and family planning service delivery, given the simultaneous risks of HIV infection and unintended pregnancy (3–7), for most women in LMICs, these services remain siloed (8).

Multipurpose prevention technology (MPT) products offer the potential to integrate sexual and reproductive health services and meet the diverse health needs of women over their reproductive lifespans (9–13). A growing body of literature indicates that a majority of women would be more interested in using an HIV-prevention method that also prevents pregnancy since preventing unintended pregnancy is often their primary concern (14–16). In a recent study from South Africa, significantly more women were interested in using the SILCS diaphragm together with a vaginal microbicide as an MPT (68%) vs. SILCS alone for contraception (17%) or a microbicide alone for HIV prevention (14%) (17). In the Share.Learn.Shape global internet survey, 83% of women preferred an HIV/STI prevention method that also prevented unintended pregnancy vs. a product for disease prevention alone (14). In the same survey, there was high interest in a range of MPT products, including on-demand, daily, and long-acting methods. Furthermore, family planning is more acceptable than disease prevention in many communities. For example, some participants in the ASPIRE trial evaluating the safety and efficacy of the dapivirine intravaginal ring (IVR) reported telling their partners that they were using a new contraceptive vs. a product for HIV prevention (18). Currently, condoms are the only available MPT, yet male condoms are not within the control of a woman, and many women risk gender-based violence by merely suggesting condom use (19). The uptake of female condoms has been limited by cost, access, and acceptability issues (including the objections of male partners) (20, 21). MPTs could help to overcome barriers to negotiating HIV prevention and adherence issues related to stigma and gender dynamics seen in trials of microbicides and oral pre-exposure prophylaxis (PrEP). Several MPTs for simultaneous HIV and pregnancy prevention are in various stages of development, however, most are likely to be many years away from market launch (22, 23). A dual prevention pill (DPP) containing PrEP co-formulated with a combined oral contraceptive (COC) is likely to be the fastest route to the introduction of a female-initiated MPT because PrEP and generic COCs are both licensed, marketed products that are widely available in many LMICs (24).

## THE PROMISE OF THE DPP

We believe that the DPP could vastly increase the number of women protected by PrEP as well as potentially increase the number of women using contraception. In the family planning arena, COCs continue to be the first-choice method for many women, despite the availability of longer-acting formulations. COCs are currently used by 151 million women worldwide (25), and over 5 million women in 15 SSA countries with a significant HIV epidemic (26). Oral PrEP, however, has had poor uptake and adherence among women to date, despite being highly effective for HIV prevention (27–29). Stigma is often cited as a reason for non-use of PrEP (30–33); women fear being regarded as HIV-positive or promiscuous if they are seen taking Truvada^®^, the same antiretroviral (ARV) drug that is used for HIV treatment. Many women have voiced concerns about the consequences PrEP will have on their sexual relationships, as PrEP use often signals mistrust and infidelity, which can potentially result in relationship dissolution or violence (33, 34). We hypothesize that the DPP has the potential to reduce the stigma associated with PrEP-only products by adding the justification of providing contraception, as many women find it easier to negotiate contraception vs. HIV/STI prevention with their partners or have a shared desire for pregnancy prevention. Furthermore, we believe women’s motivation to prevent pregnancy may drive adherence to PrEP when combined in a DPP. We estimate that by leveraging the COC market and reaching women currently using condoms or with an unmet need for contraception, between 250,000 and 1.25 million women per year in 15 SSA countries might choose to switch to the DPP, which could increase the number of women using PrEP by up to 10 times (26).

## DPP DEVELOPMENT PATHWAY

**FIGURE 1 |:**
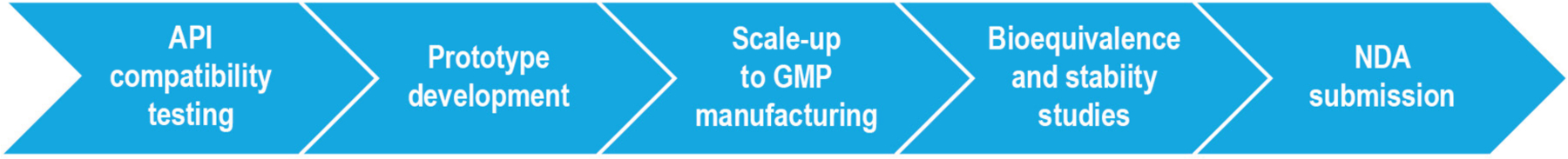
DPP Development Pathway.

The first generation DPP in development combines the active pharmaceutical ingredients (APIs) in a generic COC [150 mcg levonorgestrel (LNG), 30 mcg ethinyl estradiol (EE)] with the APIs in Truvada^®^ [300 mg tenofovir disoproxil fumarate (TDF), 200 mg emtricitabine (FTC)] or generic equivalents (35). The DPP regimen is intended to align with a 21/7 COC regimen containing 21 tablets with active COCs and PrEP, and 7 tablets containing only PrEP (vs. placebo pills in current 21/7 COC regimens). Because PrEP and COCs are already licensed, marketed products, the development pathway (Figure 1) is streamlined, requiring only a bioequivalence (BE) study rather than long, expensive Phase 3 safety and efficacy trials. In a standard BE study, healthy volunteers are enrolled in a crossover design to compare and ensure that the pharmacokinetic profile of the new drug (the DPP in this case) matches that of the reference products (Truvada and COC). Ideally, the DPP will be marketed in blister packaging to look as similar as possible to a contraceptive regimen (recognizing that Truvada is a much larger tablet than any COC). Both Viatris and the Population Council are developing DPP formulations, with potential approval as early as 2023. Given the short timeline for development, preparing for an introduction now is critical to maximizing the potential reach of the DPP.

## FRAMEWORK TO GUIDE DPP PRODUCT DEVELOPMENT AND INTRODUCTION

**FIGURE 2 |:**
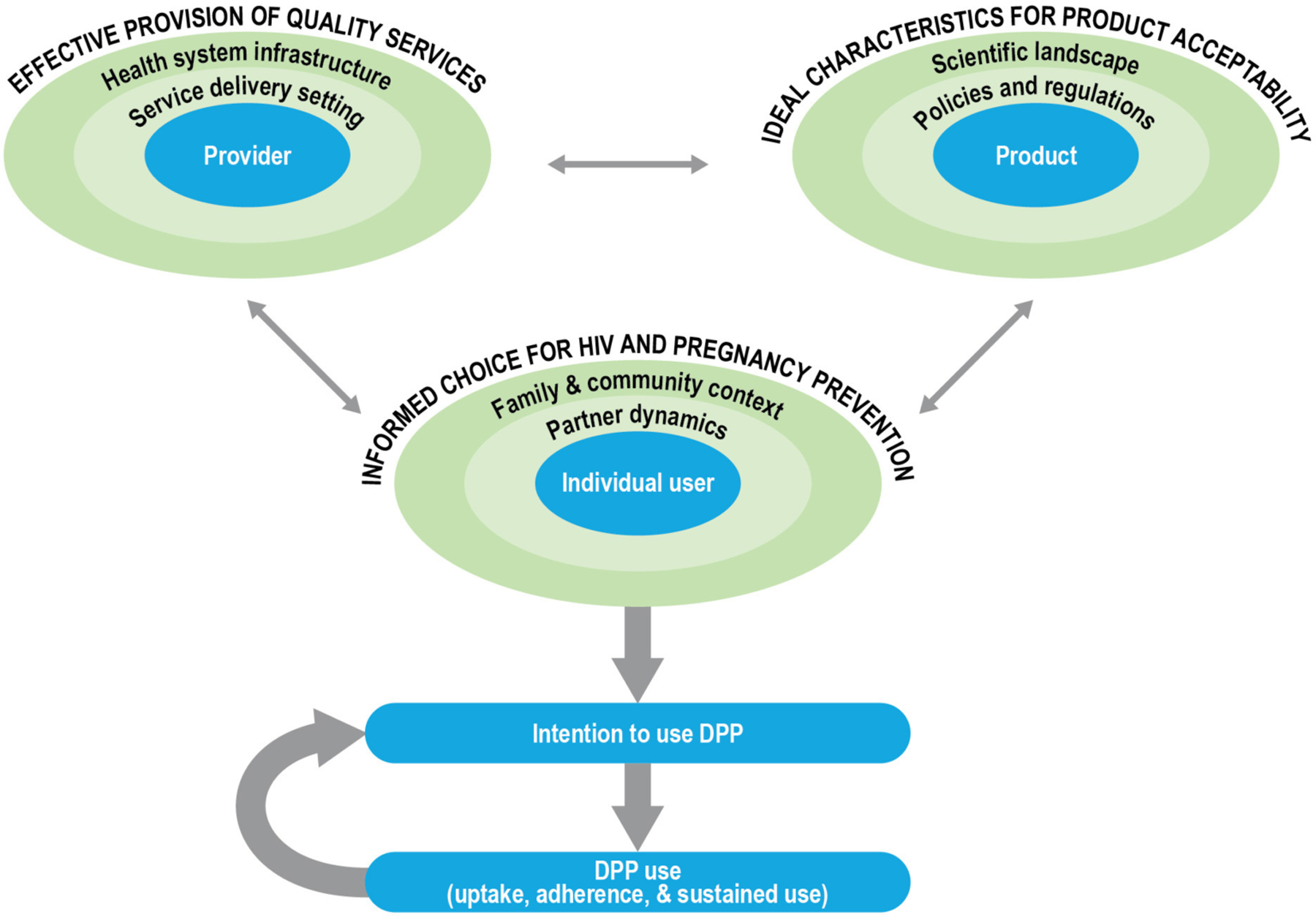
Framework for DPP development and introduction.

The development of new technology in and of itself does not imply demand, access, or use (36). The social and structural context of product provision can have an outsized influence on informed choice, product uptake, and effective and sustained use. Furthermore, there has been a growing recognition that end-user perspectives are important in the product development cycle and can help identify modifiable factors to inform formulation scientists about product attributes that may need optimization to enhance uptake, acceptability, and effective use (37–39). We propose a conceptual framework for DPP development and introduction in which user-, provider- and product-centered factors interact to influence user acceptability, intention-to-use, and, ultimately, product use (Figure 2). We used Ajzen’s Theory of Planned Behavior (TPB) to guide the outcome of interest, the behavioral intention to use the DPP, as well as the framing of user-centered factors (40). We used previous frameworks on MPT development (10), PrEP introduction (41, 42), and contraceptive development and introduction (36, 43) to articulate the provider- and product-centered factors. The user-, provider- and product-related factors are situated within their socio-ecological levels, which interact to influence the intention of women to use the DPP.

In our framework, the aim is for the ***individual user*** to be enabled ***to make informed choices for HIV and pregnancy prevention*** options in choosing the DPP. According to the TPB, attitudes, subjective norms, and perceived behavioral control of an individual influence their behavioral intentions. In our framework, the characteristics of women, as well as their partnership dynamics, and their broader family and community context shape these intentions. ***The characteristics of women*** include life stage (such as age, marital status, and parity), knowledge (such as awareness of PrEP efficacy), perceived risk of acquiring HIV or having an unintended pregnancy, attitudes (about COCs/PrEP, perceived impact of acquiring HIV or having an unintended pregnancy), and experiences (such as contraceptive/PrEP use history). ***Partner dynamics*** that are likely to influence DPP use include the type of partner(s) that women have; the HIV status of the partner, risk behaviors, awareness, and approval of the DPP or other prevention products; interpersonal power and communication within relationships; and perceived or actual impact of the DPP on sexual activity and sexual pleasure (for women and their partners). Finally, in most settings, ***family and community context*** and prevailing social and gender norms (such as community perception of fertility, childbearing, and family size), perceptions of the DPP and other prevention products, norms about contraceptive and PrEP use, and HIV-related stigma will likely influence an individual user’s intention to use the DPP.

***Provider-centered factors*** include elements that are important for ensuring the effective provision of quality services for the DPP. These include the ***healthcare providers*** and their knowledge about the DPP (such as indications, dosing regimen, counseling on side effects), attitudes, perspectives, and biases (such as frowning upon adolescent sexuality or favoring long-acting contraceptive methods for their clients), and experiences (such as the prior provision of PrEP or contraception, counseling users on adherence). Additionally, ***the service delivery setting and broader health system infrastructure*** within which healthcare providers operate are likely to influence client-provider interactions and the equitable access of women to the DPP. For example, it is critical to consider the type of training and support available to healthcare providers, their actual and perceived workload and responsibilities, product availability, and client flow at the ***service delivery setting*.** Similarly, at the ***health system*** level, product costs and financing, delivery platforms, task shifting (such as from clinicians to nurses or paramedical professionals, increased emphasis on self-care to lessen the burden on the health system), and demand generation for the DPP are just some of the key factors to consider for effective provision of the DPP.

Finally, we consider the ideal characteristics for DPP acceptability, centered around three key ***product attributes***, the dosing regimen (daily), the APIs (including contraindications and side-effect profiles of the contraceptive hormones and ARVs), and the physical properties (tablet size). Layered on top of the product attributes are the ***policies and regulations*** (such as consent laws, regulatory approvals, and financing considerations) and the broader ***scientific landscape*** (such as product effectiveness relative to other products, medical screening and monitoring, and outcomes research) that are likely to influence DPP acceptability.

The three factors outlined in the framework are interrelated and dynamic; a shift in any single factor is likely to influence the intention to use the DPP by the individual user. For example, as next-generation DPPs are considered with different formulations or with different dosing regimens, service provision and user perspectives may also shift. Further, the actual use of the DPP and the subjective evaluation of that experience by an individual, the risks and benefits, will in turn influence behavioral intentions to continue using the DPP.

## DISCUSSION

After decades of efforts to better integrate HIV and reproductive health services, the advent of novel MPTs that help women avoid unintended pregnancy and HIV may pave the way for providing more comprehensive care for individuals. The DPP offers one such potential for rapid development, introduction to the market, and expanding the method mix and choice for individual users. At the same time, a systematic and coordinated approach to evidence generation is needed across product developers, socio-behavioral researchers, program developers, end-users, healthcare providers, and key stakeholders to maximize the potential impact of the DPP (44, 45). As we lay out in the proposed framework, critical questions must be assessed for user-, provider-, and product-centered factors to facilitate the effective, efficient, and equitable introduction of the DPP.

### End-User Research

Evidence shows the importance of early engagement with potential end-users to identify facilitators and barriers to product acceptability, intention to use, product uptake, and effective use. For example, while it is assumed that combining HIV prevention with contraception will reduce the stigma associated with PrEP, empirical data will be needed to demonstrate that this is the case. Further, it will be important to ensure that rates of unintended pregnancy do not increase among COC users who switch to the DPP. For example, women taking COCs are advised to take two pills if they miss a dose; however, that is not the recommendation for PrEP and guidelines will need to be developed regarding missed doses. Counseling messages will need to be developed to position the DPP within the contraceptive method mix and ensure shared decision-making between providers and users for women to select the method that best matches their prevention priorities. Further, as with other PrEP products, women will need counseling on how to avoid STIs. Appropriate tools will need to be adapted and developed, including interactive client pre-counseling self-assessments to educate and counsel women about the anticipated side effects, risks, and benefits of the method to support effective and sustained use. Additional efforts will be needed to strategically engage male partners without diminishing women’s autonomy (33, 46–48). End-user engagement will be key to inform demand creation, branding and marketing strategies, tools to support end-users, and implementation plans.

### Engaging Providers

Providers play an instrumental role in influencing the demand, uptake, and effective use of new products. From the perspectives of reproductive health/family planning providers, the DPP or similar technologies introduced through family planning clinics could enhance integration but could also be a burden. The DPP may require additional HIV- or PrEP-related medical screening, testing, and monitoring that may be perceived to be outside of the scope of or burdensome to many family planning and primary healthcare providers. Concerns that the introduction of the DPP could influence client uptake of long-acting contraceptive methods need to be mitigated by developing guidance for healthcare providers on how to support their clients to effectively meet their HIV and pregnancy prevention goals through a shared decision-making model of counseling and provision that maximizes client autonomy and informed choice to alleviate the potential for coercion. In particular, it will be important to explore the knowledge and attitudes of providers about the DPP, which will influence the access of women and the messages regarding the DPP. Mechanisms to effectively engage and support healthcare providers, including additional training and resources, within their eco-system will be critical to informing service delivery points about the need for the DPP.

### Policy and Regulatory Considerations

Finally, to better understand how this new technology will be integrated within healthcare systems, further elucidation will be needed on product financing, market shaping and sizing, and value for money analyses. For the product developers, expanding beyond the current formulations of the DPP may help to further expand the market. Creating a co-formulated DPP with Descovy^®^ [tenofovir alafenamide (TAF) and FTC], once it is approved for use as PrEP in heterosexual women, may be more appealing to women because of its smaller size. Alternative contraceptive regimens, such as extended cycles or progestin-only pills, may simplify the DPP regimen, offer more options, including for those with contraindications to estrogen-containing products (such as post-partum women) or those who desire amenorrhea (49, 50), and address challenges in counseling messages around missed doses for COCs vs. PrEP. Incorporating end-user preferences for the ideal DPP characteristics into the product development process is likely to enhance uptake.

### Conclusion

We have laid out a broad scope of work, yet experience has shown us that asymmetric attention to any of these factors can lead to ineffective product uptake (36, 51). The proposed framework for guiding DPP development and introduction will continue to be important after the initial phases of product introduction to ensure that women can safely choose, access, and use the DPP. The DPP framework could serve as a model for the integration of future MPTs, including next-generation DPP products and other formulations like intravaginal rings and implants. Lessons learned from the DPP can pave the way for new technologies that best meet the needs of women, effectively destigmatize HIV as a general aspect of comprehensive reproductive health services, and lead to efficient integration of HIV and reproductive health services.
